# Tests for Oxalic Acid

**Published:** 1830-04-01

**Authors:** 


### Tests for Oxalic Acid. By Dr. Christison.

This substance has caused, by accident or design, more deaths, of late years, than any other individual poison. Mr. Royston, in 1814, seems to have been the first who introduced it to notice, as a poison taken, in mistake, for Epsom salts—and, strange as it may appear, the accident has become more common, in proportion as people have been put on their guard against it! The publication of its properties now makes it the common instrument of suicide, the certainty and rapidity of its operation rendering it superior to most other poisons. It is ill-adapted, however, for the purposes of murder, for obvious reasons—and yet there is one instance on record, so late as 1827, where a man tried to poison his wife, by giving her oxalic acid in gin! In appearance it closely resembles the sulphate of magnesia, for which it has been so often fatally mistaken. The taste is very different indeed, the sulphate being bitter. In determining the medico-legal tests for oxalic acid—it may be considered in two states—dissolved in water—and mixed with the contents of the stomach or bowels, in or out of the body.

1. In the former state (pure solution) its nature may be determined in the following manner:—

"The acidity of the fluid is first to be established by its effect on litmus paper. This being done the reagents might be applied at once. But it is better to neutralize the acid previously with any alkali; for then they act with greater delicacy. The remainder of the process consequently applies not only to oxalic acid itself, but also to all the soluble oxalates, which will presently be proved to be likewise active poisons.—The tests are the hydrochlorate of lime, sulphate of copper, and nitrate of silver.

*Hydrochlorate of lime* causes a white precipitate, the oxalate of lime; which is dissolved on the addition of a drop or two of nitric acid,—and is not dissolved when similarly treated with hydrochloric acid, unless the acid is used in very large proportion.

"The solubility of the oxalate of lime in nitric acid distinguishes the precipitate from the sulphate of lime, which the present test might throw down from solutions of the sulphates. The insolubility of the oxalate of lime in hydrochloric acid on the other hand distinguishes the precipitate from the tartrate, citrate, carbonate and phosphate of lime, which the test might throw down from any solution containing a salt of these acids. The last four precipitates are re-dissolved by a drop or two of hydrochloric acid; but the oxalate is not taken up till a large quantity of that acid is added.

"*Sulphate of copper* causes a bluish white precipitate, which is not re-dissolved on the addition of a few drops of hydrochloric acid. The precipitate is the oxalate of copper. It is re-dissolved by a large proportion of hydrochloric acid.

"This test does not precipitate the sulphates, hydrochlorates, nitrates, tartrates, citrates. But with the carbonates and phosphates it forms precipitates resembling the oxalate of copper. The oxalate, however, is distinguished from the carbonate and phosphate of copper by not being re-dissolved on the addition of a few drops of the hydrochloric acid.

"*Nitrate of silver* causes a dense, white precipitate, the oxalate of silver; which, when collected on a filter, dried, and heated, becomes brown on the edge, then fulminates faintly and is dispersed."*

II. By experiments made by Drs. Christison and Coindet, it appears that oxalic acid, so far as concerns the tissues of the stomach or its ordinary contents, is not altered in chemical form, and remains soluble in water. In such solution, however, a variety of soluble principles are contained which would cause abundant precipitates — two of the tests of the process—sulphate of copper and nitrate of silver—so that the oxalates of those metals could not possibly be exhibited in their characteristic forms. Hence the process for a pure solution is inapplicable to the mixtures under consideration. When antidotes are exhibited during life, changes of still greater consequence are effected. Thus magnesia and chalk are the best antidotes for oxalic acid—and if either of these be given in sufficient quantity no oxalic acid will remain in solution. The proofs of the presence of the poison must then be sought for in the solid contents of the stomach, of solid matters of vomiting. The following process for detecting the poison will apply to all the alterations which it may thus have undergone.

"The first object is to procure a solution.—If an antidote has not been given, the contents and tissues or vomited matter are to be boiled, distilled water being added if required. The acid is then to be neutralized with potass and the whole filtered.—If magnesia or chalk has been given as an antidote, the insoluble matter is to be separated by filtration and boiled for twenty minutes in a solution of carbonate of potass in 18 or 20 parts of water. A double interchange of elements takes place between a part of the carbonate of potass and a part of the oxalate of lime or magnesia; and in consequence some carbonate of lime or magnesia is thrown down, while some oxalate of potass will be found in solution. The fluid after filtration is to be neutralized with pure nitric acid.

"Oxalic acid being now in solution, whatever may have been its original state, the next step is to separate it from the animal and vegetable matter dissolved along with it. I have tried various plans for this purpose, but have found none to answer so well as precipitation with muriate of lime, so as to procure an oxalate of lime; which, after being well washed, is to be decomposed by boiling it in a solution of carbonate of potass as before. An oxalate of potass will again be found in solution. The excess of alkali is finally to be neutralized with nitric acid.

"The fluid is now to be tested with the three re-agents for the pure solution of oxalic acid."

* Christison, p. 142.

